# The longitudinal bidirectional association between cardiovascular disease and depressive symptoms among middle-aged and elderly adults: evidence from a nationwide cohort study in China

**DOI:** 10.3389/fpsyt.2025.1559092

**Published:** 2025-07-02

**Authors:** Jie He, Mingyue Liu, Ziying Zhang, Mingzhu Fang, Liang Wu, Haisheng Wu, Zhe Li

**Affiliations:** ^1^ Department of Radiology, Sir Run Run Shaw Hospital, Zhejiang University School of Medicine, Hangzhou, China; ^2^ Department of Sports Rehabilitation, Beijing Xiaotangshan Hospital, Beijing, China; ^3^ Department of Rehabilitation Medicine, The Fifth Affiliated Hospital of Zhengzhou University, Zhengzhou, China; ^4^ School of Public Health, Li Ka Shing (Faculty of Medicine), The University of Hong Kong, Hong Kong, Hong Kong SAR, China; ^5^ Henan Rehabilitation Clinical Medicine Research Center, Zhengzhou, China

**Keywords:** depression, CVD (cardio vascular disease), CLPM, longitudinal study, middle-aged and older adult

## Abstract

**Background:**

Although cardiovascular disease (CVD) and depressive symptoms frequently co-occur, their temporal directional relationship remains poorly understood. This study aimed to investigate the bidirectional association between depression and CVD in middle-aged and elderly Chinese adults through longitudinal analyses.

**Method:**

This longitudinal cohort study included 12792 participants from the China Health Retirement Longitudinal Study (CHARLS), 2015-2020. Depressive symptoms were defined as a score of 10 or higher on the 10-item Center for Epidemiological Studies Depression Scale (CES-D-10). Cardiovascular disease (CVD) was defined as physician-diagnosed coronary heart disease (CHD) and/or stroke. Cox proportional hazards models were applied to investigate the longitudinal association of baseline depressive symptoms with follow-up CVD events, as well as the association of baseline CVD status with follow-up depressive symptoms. Cross-lagged panel models were performed to evaluate bidirectional associations and strength of temporal relationships simultaneously. Effect modification by demographic and lifestyle factors was also examined to identify vulnerable populations for each directional pathway.

**Results:**

Of 12792 participants, the mean (SD) age was 60.8 (8.9) years and 6833 (53.4%) were females. After adjusting for potential confounders, baseline depressive symptoms were associated with higher risks of subsequent CVD (HR=1.55, 95% CI: 1.40-1.72), CHD (HR=1.51, 95% CI: 1.34, 1.70), and stroke (HR=1.71, 95% CI: 1.43-2.04); conversely, baseline conditions of CVD (HR=1.22, 95% CI: 1.10-1.35), CHD (HR=1.20, 95% CI: 1.08-1.33) and stroke (HR=1.43, 95% CI: 1.16-1.77) were associated with increased risk of depressive symptoms at follow-up. The cross-lagged panel analysis further confirmed the bidirectional associations over time (all *P*-values < 0.001), revealing that the standardized effect size of CVD status on depressive symptoms was greater than the effect size in the reverse direction. The directional pathway from depressive symptoms to CVD was modified by body mass index, educational level, residence, and alcohol consumption, while the reverse directional association was modified by education level and alcohol consumption.

**Conclusion:**

There is a longitudinal, bidirectional association between CVD status and depressive symptoms in mid-to-late life, with CVD status emerging as a larger driving force in these dynamic interactions. These findings suggest that targeted interventions addressing either CVD or depressive symptoms may yield reciprocal benefits over time.

## 1 Introduction

Population aging is driving a rise in age-related diseases globally, imposing substantial burdens on individuals, families, and society (1). Age-related diseases are estimated to contribute to 51.3% of the global disease burden (2). Among age-related diseases, coronary heart disease (CHD) and depression rank as the top two causes of disability in high-income countries, with this trend projected to extend globally by 2030 (3, 4). Progressive declines in the heart and vasculature systems are a natural part of aging, while a greater unexpected decline can eventually progress to cardiovascular diseases (CVD), such as CHD and stroke. Depression, as a prevalent mental disorder, is also associated with increased disability and mortality in the aging population (5). More than 350 million older adults worldwide suffer from depression, with an even larger number experiencing subclinical depressive symptoms (6). In China, a multi-center survey reported that 20.3% of older adults met diagnostic criteria for depressive disorders, including 10.2% with major depression, 4.8% with dysthymia, and 5.3% with minor depressive disorder (7). The majority of depressive symptoms remain undiagnosed or untreated, a problem particularly pronounced among middle-aged and elderly populations (8). Consequently, identifying modifiable factors for CVD and depression and implementing targeted interventions, are essential to mitigate subsequent disease burdens.

The frequent comorbidity between depressive disorder and CVD suggests an intrinsic link (9, 10), yet most previous studies were constrained by cross-sectional designs. Several studies have also examined the unidirectional longitudinal relationships between depressive symptoms and the risk of CVD (11, 12), and vice versa (13), which imply that these two conditions may be risk factors or outcomes for each other. This bidirectional interaction could be driven by shared underlying pathophysiological mechanisms or lifestyle factors, such as shared genetic substrates, metabolic and immuno-inflammatory dysregulations, physical inactivity, and unhealthy diet (14–17). Additionally, hypertension might also be a underlying mechanisms linking depression and CVD (18). However, there is still limited evidence from the same longitudinal cohort studies that simultaneously assess these associations bidirectionally. In addition, key questions remain unresolved: (1) the specific directionality of the relationships between depressive symptoms and major CVD subtypes, such as CHD and stroke, remains underexplored; (2) the demographic and lifestyle factors that may modify the distinct directional associations remain underexplored and require systematic investigation. These gaps highlight the need for tracking both incident CVD (and its major subtypes) and depressive symptoms across multiple time points to elucidate the directionality comprehensively.

To bridge this research gap, this study examined data from the community-based longitudinal survey of middle-aged and older Chinese adults, to investigate the bidirectional associations between depressive symptoms and CVD and its major subtypes in mid-to-late life. Meanwhile, the vulnerable populations and modifiable lifestyle risk factors for each potential directional association were also identified.

## 2 Methods

### 2.1 Study population

The study was based on the China Health and Retirement Longitudinal Study (CHARLS) (19). CHARLS is a nationally representative, longitudinal survey targeting Chinese adults aged 45 years and older, which employed a multistage, stratified probability-proportional-to-size sampling method across 28 provinces in China. CHARLS recruited undergraduate and graduate students as field interviewers, who received intensive training in interviewing techniques, passed qualification exams, and showed proficiency through mock rehearsals. Data collection utilized computer-assisted personal interviewing (CAPI), with in-person, face-to-face interactions. For respondents unable to participate due to health issues, cognitive limitations, or other reasons, informed proxy informants were interviewed on their behalf. Detailed methodology, questionnaire design, follow-up protocols, and quality control measures are documented in prior publications (19, 20). CHARLS has obtained ethical approval from the Biomedical Ethics Review Committee of Peking University (IRB00001052-11015), and all participants were provided with informed written consent.

**Figure 1:**
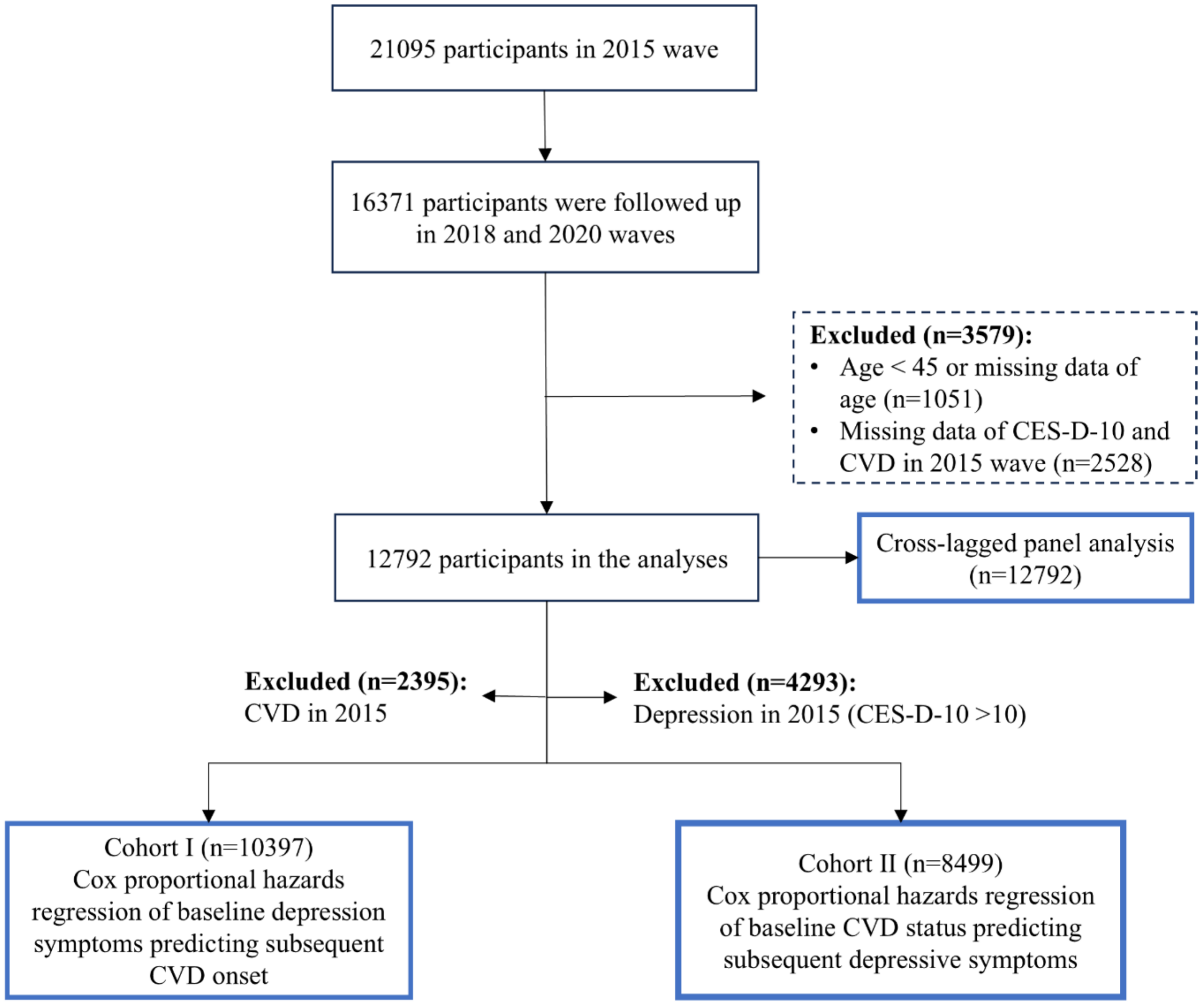
The workflow of this study.

In this study, we utilized data from the three most recent survey waves (2015, 2018, and 2020) of the CHARLS dataset, with the 2015 wave as the baseline and waves in 2018 and 2020 as the follow-up. Among the 21095 participants initially included in the 2015 wave, 16371 were successfully followed up in the 2018 and 2020 waves. We subsequently excluded 1051 participants who were either under 45 years of age or had missing age data, as well as 2528 participants with incomplete data on CES-D-10 or CVD in 2015. A final sample of 12792 participants was eligible for the cross-lagged panel analysis (**Figure 1**).

Besides, in the unidirectional longitudinal analyses, participants diagnosed with CVD at baseline (n = 2395) and those with depressive symptoms at baseline (n = 4293) were excluded, respectively. Consequently, a total of 10397 participants without initial CVD were included in Cohort I for the longitudinal association of baseline depression status with subsequent risk of incident CVD, while 8499 participants were included in Cohort II for the longitudinal association between baseline CVD status and subsequent risk of depression. The detailed participant selection process is illustrated in **Figure 1**.

### 2.2 Assessment of CVD events

Consistent with prior CHARLS-based research, CVD events were defined as the presence of CHD and/or stroke (21, 22). CHD events (heart attack, coronary heart disease, angina, congestive heart failure, or other heart problems) and stroke events were confirmed by self- or caregiver-reported clinician diagnosis, which were recorded by trained interviewers using standardized questionnaires in the face-to-face interview. This approach has been extensively applied in large-scale epidemiological studies to capture the prevalence of chronic conditions (23).

### 2.3 Measurement of depressive symptoms

In CHARLS, an individual’s depressive symptoms were evaluated using the 10-item Center for Epidemiological Studies Depression Scale (CES-D-10), a tool widely acknowledged for its robust ability to identify individuals with depressive symptoms (24). CES-D-10 has demonstrated high sensitivity in assessing depressive symptoms among older Chinese adults (25). This scale comprises 10 items, each scored on a 4-point scale ranging from 0 to 3, yielding a total score between 0 and 30, with higher scores reflecting greater severity of depressive symptoms. A threshold score of 10 was used as the cutoff for identifying depressive symptoms (26).

### 2.4 Covariates

The covariates included age, sex (male or female), body mass index [underweight (<18.5 kg/m^2^), normal weight (18.5-24.0 kg/m^2^), or overweight or obesity (≥24.0 kg/m^2^)], education level (illiterate, less than high school, or high school and above), marital status [married or other (partnered, separated, divorced, widowed, or never married)], residence (rural or urban), alcohol consumption [never, occasional (< 3 times/wk), or regular (> 3 times/wk)]], smoking status (never or ever), engagement in social activities (yes or no), personal earnings after tax (positive or non-positive), types of household cooking fuel use [clean (gas, liquefied petroleum gas, biogas, electricity, or solar energy) or solid (wood, coal, or crop residues)] and conditions (yes or no) of hypertension, diabetes and dyslipidemia.

In CHARLS, social activities over the past month were assessed through five indicators: 1) interactions with friends; 2) participation in activities such as playing Ma-jong, chess, or cards, or attending community clubs; 3) involvement in sports, social, or other clubs; 4) engagement in community-related organizations; and 5) involvement in voluntary or charity work. Hypertension was defined as a systolic blood pressure of ≥140 mmHg, diastolic pressure of ≥90 mmHg, or a self-reported history of physician-diagnosed hypertension. Diabetes was identified as a fasting glucose level ≥7.0 mmol/L, HbA1c ≥6.5%, or self-reported physician-diagnosed diabetes. Dyslipidemia was characterized by any abnormal plasma lipid level [total cholesterol (≥6.22 mmol/L), triglycerides (≥2.26 mmol/L), or LDL cholesterol (≥4.14 mmol/L)] or a self-reported history of physician-diagnosed dyslipidemia.

### 2.5 Statistical analyses

We reported baseline characteristics for total study participants, and longitudinal cohorts I and II. Continuous variables were summarized as means [standard deviation (SD)]. Categorical variables were presented with count (%).

*Longitudinal unidirectional analyses* Cox proportional hazards (PH) regression models were applied to examine the association between baseline depressive symptoms and follow-up CVD risk among individuals in Cohort I (n=10397) without baseline CVD conditions. Then Cox PH models were also used to examine the association between baseline CVD status and follow-up depressive symptoms among individuals in Cohort II (n=8499) without baseline depression status. Hazard ratios (HRs) and their corresponding 95% confidence intervals (CIs) were derived from three models: (1) Model 1 was a crude model; (2) Model 2 was a partially adjusted model, adjusting for age, sex, BMI, residence, education level, marital status, alcohol consumption, smoking status, engagement in social activities, household cooking fuel use, and personal earnings after tax; (3) Model 3 was fully adjusted model, including all covariates in Model 2 with additional adjustments for hypertension, diabetes, and dyslipidemia. The fully adjusted Model 3 was designated as the main model in this study. The multiple imputation by chained equations (MICE) was performed to impute the missing covariate data. Cox PH models used follow-up years as the time scale, with the 2015 wave date as baseline. Follow-up duration was calculated from 2015 to reported CVD/depression events or censoring (loss to follow-up, death, or the 2020 wave end). Due to the wave-based longitudinal design of CHARLS, event times were interval-censored, so Cox-derived HRs are approximate and require cautious interpretation.

*Cross-lagged panel analysis* To further validate the bidirectional association from two longitudinal unidirectional analyses, a cross-lagged panel model (CLPM) was used among the total participants (n=12792). CLPM analysis explored the reciprocal influence of the two factors over time while adjusting for their baseline levels, enabling an evaluation of the strength of their temporal relationship. Previous studies have demonstrated that CLPM approach provided more robust evidence for determining the temporal sequence between the two variables (27, 28). We established a fully adjusted CLPM, adjusting for baseline covariates as the Cox Model 3. Full information maximum likelihood estimation with robust (Huber-White) standard errors was applied to address the incomplete data on covariates.

*Stratification analysis* We further conducted subgroup analyses to examine whether the different longitudinal unidirectional associations of depressive symptoms with CVD were modified by age (45-59 years vs.≥60 years), sex (male vs. female), BMI group (normal weight vs. underweight/overweight or obesity), educational level (illiterate vs. less than high school/high school and above), marital status (married vs. other), residence (urban vs. rural), alcohol consumption (never vs. occasional/frequent), smoking history (never vs. ever), and social activity engagement (no vs. yes). Stratification analyses were performed based on the fully adjusted Cox PH model. We also performed stratification analyses for the longitudinal unidirectional association between depressive symptoms and CVD subtypes (i.e., CHD and stroke). The z-test was performed to determine whether the estimated effects differed significantly between subgroups (29). Additionally, for the CLPM analysis, subgroup analyses by the key demographic characteristics (i.e., age and sex) were also performed.

*Sensitivity analysis*. Several sensitivity analyses were performed. First, we confined the Cox PH model and CLPM analysis among participants without missing covariate data, aiming to examine whether the data imputation process for missing covariate data potentially introduced bias and whether the corresponding results of depression-CVD association were robust. Second, previous studies have reported ambient pollution exposure is associated with both depressive symptoms and CVD risk (30, 31). To account for the potential confounding effects of air pollution, we additionally included the long-term ambient fine particulate matter (PM_2.5_) exposure in the main Cox PH model and CLPM analysis. The procedure of PM_2.5_ exposure assessment was consistent with our previous publication (30), which was based on the high-resolution Tracking Air Pollution (TAP) dataset (http://tapdata.org.cn) and geocoded residential regionalization of each participant. The moving average of PM_2.5_ concentrations for 1 year before the baseline was used as the proxy for the long-term exposure level.

All statistical analyses were performed using R software (version 4.3.1). A two-sided *P*-value <0.05 was considered indicative of statistical significance.

## 3 Results

### 3.1 Participant characteristics

**Table 1** reported the baseline characteristics of the study population. A nationwide sample of 12792 middle-aged and older adults was finally included in the analysis, of whom 53.4% were females, 64.8% were rural residents and 25.6% had no formal education. The mean (SD) age of the total participants was 60.8 (8.9) years. There were 2,000 (15.63%), 39 (0.30%), 1 (0.008%), 117 (0.91%) and 23 (0.18%) missing values for BMI, alcohol consumption, smoking history, personal earning and household cooking fuel, respectively (**Table 1**). Collectively, there were 2126 (16.6%) participants with missing data.

**Table 1:** Baseline characteristics of study subjects.

Characteristics	Total participants	Cohort I (analytical sample for baseline depressive symptoms and subsequent CVD)	Cohort II (analytical sample for baseline CVD conditions and subsequent depressive symptoms)
No.	12792	10397	8499
Age, year, (mean ± SD)	60.8 ± 8.9	60.3 ± 8.9	60.6 ± 9.0
Sex, *n* (%)			
Male	5959 (46.6)	4991 (48.0)	4453 (52.4)
Female	6833 (53.4)	5406 (52.0)	4046 (47.6)
Education, *n* (%)			
Illiterate	3274 (25.6)	2639 (25.4)	1852 (21.8)
Less than high school	8028 (62.8)	6596 (63.4)	5429 (63.9)
High school and above	1490 (11.6)	1162 (11.2)	1218 (14.3)
Residence, *n* (%)			
Urban	4497 (35.2)	3534 (34.0)	3333 (39.2)
Rural	8295 (64.8)	6863 (66.0)	5166 (60.8)
Marital status, *n* (%)			
Married	10545 (82.4)	8610 (82.8)	7212 (84.9)
Other	2247 (17.6)	1787 (17.2)	1287 (15.1)
BMI, kg/m^2^, (mean ± SD)	23.9 ± 3.9	23.7 ± 3.7	24.0 ± 3.85
BMI category, *n* (%) ^a^			
Underweight	562 (4.4)	466 (4.5)	320 (3.8)
Normal weight	5211 (40.7)	4436 (42.7)	3386 (39.8)
Overweight or obesity	5019 (39.2)	3865 (37.2)	3430 (40.4)
Alcohol consumption, *n* (%) ^a^			
Never	8301 (64.9)	6598 (63.5)	5238 (61.6)
Occasional (< 3 times/wk)	2535 (19.8)	2094 (20.1)	1813 (21.3)
Frequent (> 3 times/wk)	1917 (15.0)	1670 (16.1)	1430 (16.8)
Smoking history, *n* (%) ^a^			
Never	7291 (57.0)	5880 (56.6)	4553 (53.6)
Ever	5500 (43.0)	4516 (43.4)	3946 (46.4)
Social activity engagement, *n* (%)			
No	6603 (51.6)	5430 (52.2)	4128 (48.6)
Yes	6189 (48.4)	4967 (47.8)	4371 (51.4)
Personal earning, *n* (%) ^a^			
Non-positive	10089 (78.9)	8039 (77.3)	6423 (75.6)
Positive	2586 (20.2)	2258 (21.7)	1987 (23.4)
Household cooking fuel, *n* (%) ^a^			
Cleaner	7283 (56.9)	5984 (57.6)	5215 (61.4)
Solid	5486 (42.9)	4394 (42.3)	3268 (38.5)
Hypertension, *n* (%)			
No	7551 (59.0)	6608 (63.6)	5097 (60.0)
Yes	5241 (41.0)	3789 (36.4)	3402 (40.0)
Diabetes, *n* (%)			
No	10569 (82.6)	8807 (84.7)	7079 (83.3)
Yes	2223 (17.4)	1590 (15.3)	1420 (16.7)
Dyslipidemia, *n* (%)			
No	7359 (57.5)	6205 (59.7)	4903 (57.7)
Yes	5433 (42.5)	4192 (40.3)	3596 (42.3)

a Missing data: 2000 for BMI, 39 for alcohol consumption, 1 for smoking history, 117 for personal earning, and 23 for household cooking fuel.

BMI, body mass index; CVD, cardiovascular disease.

In the longitudinal Cohort I including 10397 participants without baseline CVD conditions, 1908, 1389 and 677 participants developed new-onset CVD, CHD, and stroke, respectively (**Table 2**). Furthermore, in the longitudinal Cohort II comprising 8499 participants without baseline depressive symptoms, 3162 participants experienced new-onset depressive symptoms during the follow-up (**Table 3**). More baseline characteristics for Cohorts I and II are presented in **Table 1**.

**Table 2:** Hazard ratios (HRs) and 95% confidence intervals (CIs) for new-onset CVD associated with baseline depressive symptoms (n = 10397).

Outcome	No. of event	Unadjusted model		Partially adjusted model ^a^		Fully adjusted model ^b^	
		HR (95% CI)	*P*	HR (95% CI)	*P*	HR (95% CI)	*P*
CVD	1908	1.63 (1.49, 1.79)	< 0.001	1.56 (1.41, 1.73)	< 0.001	1.55 (1.40, 1.72)	< 0.001
CHD	1389	1.61 (1.45, 1.79)	< 0.001	1.52 (1.35, 1.72)	< 0.001	1.51 (1.34, 1.70)	< 0.001
Stroke	677	1.75 (1.51, 2.04)	< 0.001	1.73 (1.45, 2.06)	< 0.001	1.71 (1.43, 2.04)	< 0.001

HR, hazard ratio; CI, confidence interval.

^a^Partially adjusted model was adjusted for age, sex, BMI, residence, education level, marital status, alcohol consumption, smoking status, engagement in social activities, household fuel use, and personal earnings after tax.

^e^Fully adjusted model was adjusted as the partially adjusted model with further adjustments for hypertension, diabetes, and dyslipidemia.

**Table 3:** Hazard ratios (HRs) and 95% confidence intervals (CIs) for new-onset depression associated with baseline CVD conditions (n = 8499).

Baseline condition	No. of new-onset depression	Unadjusted model		Partially adjusted model ^a^		Fully adjusted model ^b^	
		HR (95% CI)	*P*	HR (95% CI)	*P*	HR (95% CI)	*P*
CVD	3162	1.22 (1.11, 1.33)	< 0.001	1.24 (1.12, 1.37)	< 0.001	1.22 (1.10, 1.35)	< 0.001
CHD	3162	1.20 (1.09, 1.32)	< 0.001	1.22 (1.10, 1.35)	< 0.001	1.20 (1.08, 1.33)	0.001
Stroke	3162	1.39 (1.15, 1.69)	< 0.001	1.47 (1.19, 1.82)	< 0.001	1.43 (1.16, 1.77)	0.001

CHD, coronary heart disease; CI, confidence interval; CVD, cardiovascular disease; HR, hazard ratio.

^a^Partially adjusted model was adjusted for age, sex, BMI, residence, education level, marital status, alcohol consumption, smoking status, engagement in social activities, household fuel use, and personal earnings after tax.

^e^Fully adjusted model was adjusted as the partially adjusted model with further adjustments for hypertension, diabetes, and dyslipidemia.

### 3.2 Influence of baseline depressive symptoms on follow-up CVD

The Cox PH regression analysis of 10397 participants in Cohort I revealed that baseline depressive symptoms were significantly associated with an elevated risk of subsequent CVD (HR = 1.63, 95% CI: 1.49–1.79, *P* < 0.001; **Table 2**). This relationship remained significant after adjusting for potential confounders in the partially adjusted model (HR = 1.56, 95% CI: 1.41–1.73, *P* < 0.001; **Table 2**). Even with further adjustments for hypertension, diabetes, and dyslipidemia in the fully adjusted model, the association remained robust (HR = 1.55, 95% CI: 1.40–1.72, *P* < 0.001; **Table 2**).

When examining the CVD subtypes of CHD and stroke, baseline depressive symptoms were also significantly linked to higher risks in all three models across different adjustment levels. In the fully adjusted model, baseline depressive symptoms were associated with a 51% increased risk of CHD (HR = 1.51, 95% CI: 1.34–1.70, *P* < 0.001; **Table 2**) and a 71% increased risk of stroke (HR = 1.71, 95% CI: 1.43–2.04, *P* < 0.001; **Table 2**) during the follow-up period.

### 3.3 Influence of baseline CVD status on follow-up depressive symptoms

The Cox PH regression analysis of 8499 participants in Cohort II revealed that baseline CVD status was significantly associated with an elevated risk of subsequent depressive symptoms in the crude model (HR = 1.22, 95% CI: 1.11–1.33, *P* < 0.001; **Table 3**). This relationship persisted after adjusting for potential confounders in the partially adjusted model (HR = 1.24, 95% CI: 1.12–1.37, *P* < 0.001; **Table 2**) and fully adjusted model (HR = 1.22, 95% CI: 1.10–1.35, *P* < 0.001; **Table 3**).

All models with different adjustment levels also indicated consistent associations of CHD and stroke at baseline and subsequent risk of depressive symptoms. In the fully adjusted model, baseline CHD status was associated with a 20% increased risk of subsequent depressive symptoms (HR = 1.20, 95% CI: 1.08–1.33, *P* = 0.001; **Table 3**), while baseline stroke was associated with a 43% increased risk of subsequent depressive symptoms (HR = 1.43, 95% CI: 1.16–1.77, *P* = 0.001; **Table 3**).

### 3.4 Longitudinal bidirectional association between CVD and depressive symptoms

**Figure 2:**
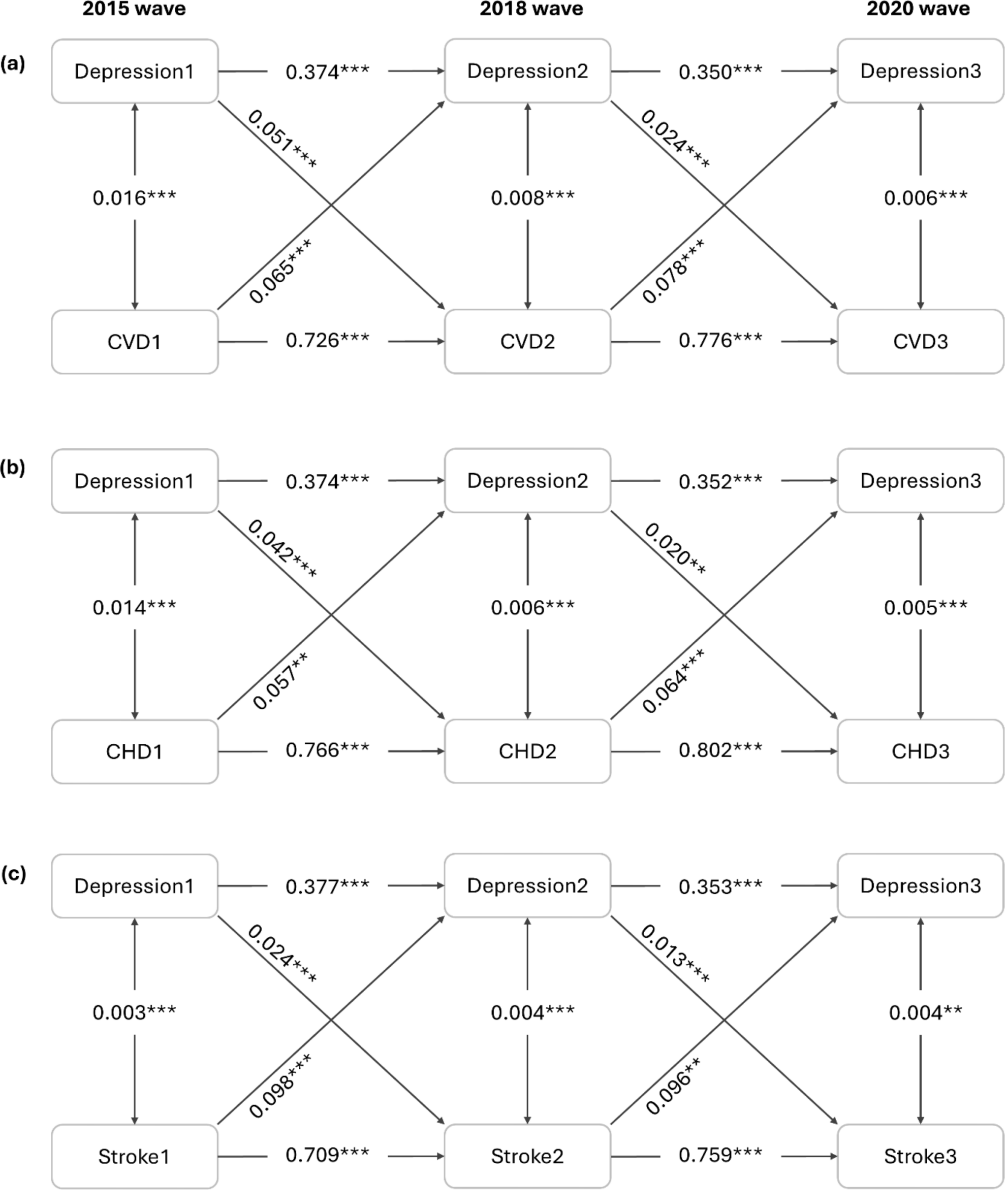
Cross-lagged panel analysis (n=12792). All coefficients are standardized. Symbol ** indicates 0.001 ≤ *P* < 0.01; Symbol *** indicates *P* < 0.001. The model was adjusted for age, sex, BMI, residence, education level, marital status, alcohol consumption, smoking status, engagement in social activities, household fuel use, personal earnings after tax, hypertension, diabetes and dyslipidemia. CHD, coronary heart disease; CVD, cardiovascular disease.

A three-wave CLPM was further established to examine the bidirectionality simultaneously. **Figure 2** reports the standardized coefficients after fully adjusting for inter-wave correlations and potential confounders. The results show that depressive symptoms at the 2015 wave were significantly associated with CVD risk at the 2018 wave (β=0.051, *P*<0.001), and depressive symptoms at the 2018 wave were still significantly associated with CVD risk at the 2020 wave (β=0.024, *P*<0.001). There were also a significant association between CVD status at the 2015 wave and depressive symptoms at the 2018 wave (β=0.065, *P*<0.001), and a significant association between CVD status at the 2018 wave and depressive symptoms at the 2020 wave (β=0.078, *P*<0.001). By comparing the standardized cross-lagged path coefficients, we found that the direction from baseline CVD status to later depressive symptoms was relatively stronger than the one from baseline depressive symptoms to subsequent CVD risk. Additionally, significant longitudinal bidirectional associations of depressive symptoms with CHD and stroke were also observed by CLPM.

### 3.5 Effect modification of the longitudinal unidirectional analysis

**Table 4** shows the stratification analysis results for different subgroups evaluating whether demographic and lifestyle factors modified the effects of baseline depressive symptoms on subsequent risks of CVD and its subtypes. The depression-CVD associations were more pronounced among individuals with abnormal weight, no formal education, rural residency, and frequent alcohol consumption. Similarly, the stratification results for depression-CHD associations aligned with those observed for depression-CVD associations. In contrast, the depression-stroke relationship was more prominent among older adults, females, and individuals with abnormal weight, a history of smoking, and no engagement in social activities.

**Table 4:** Stratification analysis for the directional associations between baseline depressive symptoms and new-onset CVD.

Stratification	Depression → CVD		Depression → CHD		Depression → Stroke	
	HR (95% CI)	*P* for effect modification	HR (95% CI)	*P* for effect modification	HR (95% CI)	*P* for effect modification
Age (years)						
45-59	1.49 (1.30, 1.69)	Ref	1.50 (1.23, 1.82)	Ref	1.55 (1.25, 1.92)	Ref
≥60	1.65 (1.40, 1.95)	0.125	1.50 (1.29, 1.75)	0.964	2.09 (1.54, 2.82)	**0.005**
Sex						
Male	1.49 (1.26, 1.76)	Ref	1.63 (1.34, 1.99)	Ref	1.41 (1.09, 1.83)	Ref
Female	1.59 (1.39, 1.82)	0.343	1.43 (1.23, 1.67)	0.121	2.05 (1.61, 2.62)	**< 0.001**
BMI						
Underweight	2.07 (1.35, 3.18)	0.157	1.86 (1.15, 3.01)	0.450	3.01 (1.20, 7.53)	**0.035**
Normal weight	1.35 (1.16, 1.56)	Ref	1.32 (1.11, 1.58)	Ref	1.42 (1.10, 1.83)	Ref
Overweight or obesity	1.74 (1.49, 2.03)	**< 0.001**	1.67 (1.39, 1.99)	**0.007**	1.99 (1.54, 2.57)	**0.002**
Education level						
Illiterate	1.70 (1.41, 2.04)	Ref	1.58 (1.27, 1.95)	Ref	1.97 (1.43, 2.72)	Ref
Less than high school	1.51 (1.32, 1.72)	0.107	1.51 (1.29, 1.76)	0.618	1.61 (1.29, 2.02)	0.073
High school and above	1.21 (0.81, 1.79)	**0.028**	1.05 (0.66, 1.67)	**0.044**	1.64 (0.84, 3.20)	0.380
Marital status						
Other	1.56 (1.24, 1.97)	Ref	1.46 (1.11, 1.93)	Ref	1.80 (1.20, 2.69)	Ref
Married	1.54 (1.38, 1.73)	0.890	1.50 (1.31, 1.72)	0.804	1.70 (1.40, 2.07)	0.675
Residence						
Urban	1.33 (1.10, 1.61)	Ref	1.27 (1.02, 1.59)	Ref	1.51 (1.09, 2.08)	Ref
Rural	1.65 (1.46, 1.86)	**0.007**	1.62 (1.40, 1.87)	**0.010**	1.81 (1.46, 2.23)	0.127
Alcohol consumption						
Never	1.50 (1.33, 1.70)	Ref	1.44 (1.25, 1.66)	Ref	1.75 (1.42, 2.15)	Ref
Occasional (< 3 times/wk)	1.42 (1.05, 1.92)	0.603	1.41 (0.99, 2.00)	0.870	1.33 (0.80, 2.22)	0.141
Frequent (> 3 times/wk)	1.95 (1.51, 2.52)	**0.002**	1.98 (1.48, 2.66)	**0.001**	1.86 (1.21, 2.87)	0.633
Smoking history						
Never	1.50 (1.27, 1.77)	Ref	1.48 (1.27, 1.72)	Ref	1.50 (1.16, 1.95)	Ref
Ever	1.59 (1.39, 1.81)	0.419	1.55 (1.27, 1.89)	0.590	1.93 (1.52, 2.45)	**0.018**
Social activity engagement						
No	1.52 (1.32, 1.75)	Ref	1.41 (1.20, 1.66)	Ref	2.01 (1.58, 2.55)	Ref
Yes	1.61 (1.38, 1.87)	0.409	1.63 (1.37, 1.94)	0.078	1.48 (1.13, 1.93)	**0.003**

a *P* values from the z-test were used to assess effect modification. The *P* values in bold are <0.05.

CHD, coronary heart disease; CI, confidence interval; CVD, cardiovascular disease; HR, hazard ratio.

The bold values represent the P for effect modification < 0.05.

**Table 5** presents the stratification analysis results for different subgroups exploring whether demographic and lifestyle factors modified the associations of baseline CVD status with follow-up depressive symptoms. The CVD-depression associations were more pronounced among individuals with no formal education and regular alcohol consumption. However, there was no consistent increasing or decreasing trend of the depression-CHD relationships within categories of demographic and lifestyle factors. Notably, the depression-stroke relationships were stronger among older adults, and individuals with no formal education, urban residency, and regular alcohol consumption.

**Table 5:** Stratification analysis for the directional associations between baseline CVD condition and subsequent depressive symptoms.

Stratification	CVD → Depression		CHD → Depression		Stroke → Depression	
	HR (95% CI)	*P* for effect modification ^a^	HR (95% CI)	*P* for effect modification ^a^	HR (95% CI)	*P* for effect modification ^a^
Age (years)						
45-59	1.16 (0.98, 1.37)	Ref	1.18 (0.99, 1.40)	Ref	0.94 (0.60, 1.46)	Ref
≥60	1.26 (1.11, 1.43)	0.331	1.21 (1.06, 1.38)	0.779	1.70 (1.33, 2.17)	**0.003**
Sex						
Male	1.30 (1.11, 1.53)	Ref	1.25 (1.06, 1.48)	Ref	1.50 (1.13, 2.00)	Ref
Female	1.17 (1.02, 1.33)	0.193	1.17 (1.02, 1.34)	0.462	1.32 (0.96, 1.82)	0.409
BMI						
Underweight	1.30 (0.78, 2.16)	0.784	1.27 (0.74, 2.18)	0.907	1.84 (0.66, 5.11)	0.187
Normal weight	1.23 (1.05, 1.44)	Ref	1.23 (1.04, 1.46)	Ref	1.11 (0.78, 1.58)	Ref
Overweight or obesity	1.21 (1.05, 1.38)	0.847	1.17 (1.01, 1.34)	0.549	1.67 (1.26, 2.21)	0.015
Education level						
Illiterate	1.37 (1.13, 1.66)	Ref	1.29 (1.05, 1.58)	Ref	1.75 (1.20, 2.54)	Ref
Less than high school	1.12 (0.99, 1.28)	**0.033**	1.12 (0.98, 1.29)	0.198	1.24 (0.94, 1.65)	**0.035**
High school and above	1.41 (1.03, 1.92)	0.859	1.39 (1.02, 1.91)	0.574	1.84 (0.93, 3.65)	0.810
Marital status						
Other	1.26 (0.98, 1.62)	Ref	1.22 (0.93, 1.60)	Ref	1.51 (0.91, 2.49)	Ref
Married	1.21 (1.08, 1.35)	0.713	1.19 (1.06, 1.34)	0.840	1.40 (1.11, 1.78)	0.708
Residence						
Urban	1.16 (0.98, 1.38)	Ref	1.11 (0.93, 1.32)	Ref	1.79 (1.28, 2.51)	Ref
Rural	1.25 (1.11, 1.42)	0.391	1.26 (1.10, 1.43)	0.188	1.26 (0.96, 1.66)	**0.018**
Alcohol consumption						
Never	1.19 (1.05, 1.34)	Ref	1.14 (1.01, 1.30)	Ref	1.53 (1.20, 1.96)	Ref
Occasional (< 3 times/wk)	1.19 (0.94, 1.50)	0.978	1.29 (1.02, 1.64)	0.285	0.67 (0.34, 1.30)	**0.017**
Frequent (> 3 times/wk)	1.61 (1.20, 2.16)	**0.009**	1.46 (1.07, 2.00)	0.064	2.79 (1.54, 5.04)	**< 0.001**
Smoking history						
Never	1.18 (1.04, 1.35)	Ref	1.19 (1.04, 1.36)	Ref	1.26 (0.92, 1.72)	Ref
Ever	1.28 (1.09, 1.50)	0.353	1.22 (1.03, 1.45)	0.762	1.59 (1.18, 2.13)	0.133
Social activity engagement						
No	1.18 (1.02, 1.36)	Ref	1.13 (0.97, 1.31)	Ref	1.46 (1.10, 1.95)	Ref
Yes	1.25 (1.08, 1.44)	0.507	1.26 (1.08, 1.46)	0.233	1.40 (1.02, 1.93)	0.772

a *P* values from the z-test were used to assess effect modification. The *P* values in bold are <0.05.

CHD, coronary heart disease; CI, confidence interval; CVD, cardiovascular disease; HR, hazard ratio.

The bold values represent the P for effect modification < 0.05.

### 3.6 Subgroup analysis of cross-lagged panel model

**Supplementary Figures S1**, **S2** show the subgroup analysis of CLPM by age and sex. The longitudinal bidirectional associations between depressive symptoms and CVD remain robust among both middle-aged adults (40-59 years) years and older adults (≥60 years). Additionally, when fitting CLPM separately by sex, the results of females and males were similar and consistent with the main results.

### 3.7 Sensitivity analysis

Results from the 10666 participants without missing data show a consistent pattern with the main results (**Supplementary Tables S1**, **S2**, **Supplementary Figure S3**), suggesting the data imputation process was less likely to introduce bias and the main results from multiple imputation data were robust. When additionally accounting for the confounding effects of long-term PM_2.5_ exposure, the bidirectional depressive symptoms-CVD association remained robust (**Supplementary Tables S3**, **S4**, **Supplementary Figure S4**).

## 4 Discussion

In this nationally representative, population-based longitudinal cohort of community-dwelling middle-aged and elderly adults in China, we identified longitudinal bidirectional associations between CVD status and depressive symptoms. The standardized cross-lagged path coefficients indicated that the direction from baseline CVD status to follow-up depressive symptoms was relatively stronger than the one from baseline depressive symptoms to subsequent CVD events. Furthermore, individuals with abnormal weight, no formal education, rural residency, and frequent alcohol consumption were identified as vulnerable populations for the pathway from baseline depressive symptoms to subsequent CVD risk. Participants with no formal education and frequent alcohol consumption were particularly vulnerable to the path from baseline CVD status to subsequent depressive symptoms.

While extensive cross-sectional studies have suggested a link between CVD and depressive symptoms, they are inherently limited in determining the directionality of this link (11–13). Several longitudinal studies have also been conducted, but most focused on unidirectional associations of depressive symptoms with later CVD risk (11, 12, 32). For instance, a community-based longitudinal study among 3086 young adults aged 18-30 in the US revealed that depressive symptoms were linked to poorer cardiovascular health during a follow-up period over 20 years (32); a larger pooled analysis of longitudinal data on 162036 participants (mean age = 63 years) from the Emerging Risk Factors Collaboration and the UK Biobank provided robust evidence of the harmful effects of depressive symptoms on CVD and its two major subtypes (CHD and stroke) among middle-aged and older adults (12). This study built upon and extended the findings of previous research. Specifically, our research not only corroborated these findings on the directional path from depressive symptoms and later CVD risk, but also identified a reverse directional path from CVD status to later depression. Evidence on the longitudinal association of baseline CVD health with later depressive symptoms has been scarce; nevertheless, a recent longitudinal study reported an influence of baseline stroke status on the subsequent development of depressive symptoms, which strengthened our findings (13). These findings underscore the importance of addressing both CVD and depressive symptoms in clinical and public health interventions among middle-aged and older adults, given their reciprocal and dynamic relationship.

Furthermore, we incorporated a cross-lagged panel model to validate the bidirectionality between depressive symptoms and CVD, by concurrently evaluating the bidirectional association over time. The results supported that these two factors are reciprocally linked, and further revealed that CVD status was the primary driving force in these interactions. Although no prior study has directly assessed this bi-directionality and its relative strength, evidence from existing research provides supportive clues. For instance, one study reported that slower gait speed may play a dominant role in the gait-cognition association, suggesting the potentially pivotal influence of physical health on the interaction between physical function and brain health (33). Additionally, a recent CHARLS-based study, revealed a bidirectional relationship between physical multimorbidity and depressive symptoms during 2011-2015, providing similar insights to our study (34). Furthermore, two recent systematic reviews have also summarized the potential of the bidirectional relationship between depressive symptoms and cardiovascular health (17, 35), and our study contributed new evidence to support this bi-directionality. Our results highlighted the dynamic interplay between physical and psychological health, warranting further to develop integrated intervention strategies targeting both domains.

There have been proposed mechanisms for unhealthy lifestyles and biological dysregulation underlying the bidirectional relationship between depressive symptoms and CVD (17, 35). On the one hand, individuals experiencing depressive symptoms face elevated risks of CVD and poor cardiovascular health, which can be attributed to a greater propensity for engaging in high-risk behaviors, including smoking, unhealthy diets, reduced physical activity, and excessive alcohol consumption (17, 36–38). For smoking, substantial evidence has been accumulated demonstrating its impact on human vascular biology and function, with a focus on the main drivers of adverse cardiovascular effects including endothelial dysfunction, inflammation, and oxidative stress (39). Unhealthy diets, such as a high intake of sugar and trans fatty acids (40, 41), can lead to dyslipidemia and insulin resistance (42), and consequently significantly increase CVD risk. Reduced physical activity is associated with lower levels of endorphins and other mood-boosting neurotransmitters (43), while simultaneously contributing to weight gain and poor cardiovascular fitness (44). Additionally, excessive alcohol consumption has been demonstrated to be relevant to both depression and CVD (45). On the other hand, several biological dysregulations have linked depression with CVD, including autonomic dysregulation, hypothalamic-pituitary-adrenal (HPA) axis dysregulation, metabolic dysregulation, and immuno-inflammatory (17). Autonomic imbalances can lead to abnormal heart rate variability and increased blood pressure variability, and ultimately contribute to the development of CVD (46). Moreover, autonomic imbalances are also associated with altered mood states typical of depression (47). Regarding HPA axis dysregulation, it serves as a crucial node within the brain’s stress circuit and is believed to play a role in depression (48, 49); meanwhile, it is hypothesized that the increased activation of the HPA axis plays a significant etiological role in the development of the metabolic syndrome and its consequences such as CVD (50). Metabolic dysregulations, such as those associated with abdominal obesity and dyslipidemia, have also been implicated in the development and progression of depression and CVD (17). A recent large-scale study has demonstrated that depression is associated with lipid dysregulation (49), which is a known risk factor for CVD (51). Higher systemic inflammatory responses can be detected in patients with depressive symptoms, which are also recognized as risk factors for incident CVD (52, 53).

Identifying vulnerable populations and modifiable risk factors is crucial for informing preventive and interventional strategies. Our study conducted stratification analyses to examine the effect modification by various demographic and lifestyle factors. For the pathway from baseline depressive symptoms to subsequent CVD, we found that the associations were more pronounced in participants with abnormal weight, no formal education, rural residency, and frequent alcohol consumption. Overweight and obesity, along with higher alcohol consumption, have been well-documented as significant contributors to CVD onset (45, 54). Conversely, higher education levels appear to offer a protective effect against CVD risk (55), possibly because individuals with advanced education are more likely to manage their mental health and cardiovascular health proactively. Participants residing in urban areas generally exhibit more favorable cardiovascular risk profiles compared to rural residents (56), which may explain the stronger association between depressive symptoms and incident CVD among rural populations. For the reverse pathway from baseline CVD status to subsequent depressive symptoms, the associations were modified by education level and alcohol consumption. Participants without formal education were more likely to develop depression compared to those with moderate education levels, aligning with prior evidence suggesting that literacy education can serve as a treatment for depressive patients (57). Furthermore, heavy alcohol consumption, a well-known detrimental lifestyle factor, has been linked to an elevated risk of depression in middle-aged and older adults (58). These findings underscore the importance of targeted interventions aimed at modifiable lifestyle factors, particularly in vulnerable subgroups such as rural residents, individuals with lower education levels, and those with unhealthy weight or drinking habits.

This study has several strengths. First, it not only replicated prior findings on the longitudinal relationship between CVD status and depressive symptoms but also extended the understanding by revealing their bidirectional association and identified the CVD status as the dominant contributor to this dynamic reciprocal interplay. Second, by exploring the effect of demographic and lifestyle factors, this study offered insights into the potential management and prevention strategies in each directional path. Furthermore, leveraging a nationwide longitudinal cohort and adjusting for a comprehensive range of potential confounders enhances the robustness of the findings. This methodological rigor minimized bias and improved the generalizability of the results to the broader population of middle-aged and elderly Chinese adults.

Nevertheless, this study has several limitations that should be acknowledged. First, in this observational study, we can only reveal associations between CVD and depressive symptoms rather than establishing causality. Confounding factors and the possibility of reverse causation might not be adequately controlled in this observational study design, highlighting the need for future Mendelian randomization (MR) studies, and experimental or natural experiment research to strengthen causal inference. Second, this study relied on self-reported depressive symptoms and CVD. The incidence of CVD might be underreported due to potential underreporting and a lack of medical examinations. Nevertheless, in CHARLS, health data on depressive symptoms and chronic CVD conditions were collected by trained interviewers utilizing standardized questionnaires that were harmonized with international aging surveys like the Health and Retirement Study (HRS) and the Survey of Health, Ageing and Retirement in Europe (SHARE). Self-reports continue to be a valuable instrument for assessing the prevalence of chronic diseases such as CVD in large-scale surveys where direct clinical evaluations are not practicable; the validity of self-reports has been verified in population-based aging longitudinal surveys (59), and the approach of self-reports has been extensively employed in prior epidemiological studies (21, 30, 60, 61). Third, due to the absence of medical diagnosis records and International Classification of Diseases (ICD) codes, CVD and its subtypes could not be clinically verified. Fourth, a limitation regarding generalizability exists as our study focused on community-dwelling middle-aged and elderly people, who differ from those in institutional settings like nursing homes or hospital patients in living conditions, healthcare access, and health profiles. Hence, our findings may not apply equally to those non-community-dwelling middle-aged and older adults, highlighting the need for future studies to cover other specific populations for broader generalizability. Fifth, this study used data from the latest three survey waves (2015, 2018, and 2020), restricting the follow-up period to five years. Longer follow-up studies are required to validate and extend our results. Finally, there is the potential for biased associations arising from residual or unmeasured confounders (e.g., social support). Nevertheless, we adjusted for a wide range of covariates informed by prior studies, including potential confounding from ambient air pollution exposure (30, 31).

## 5 Conclusion

In summary, our study provides novel evidence of a longitudinal bidirectional relationship between CVD and depressive symptoms among middle-aged and elderly adults in China. The identified bidirectional association suggests that interventions targeting either CVD or depressive symptoms could yield reciprocal benefits over time, contributing to healthier aging trajectories. Additionally, larger standardized cross-lagged coefficients indicate that CVD status may serve as the primary driving force in this dynamic interplay. Further studies are warranted to validate these findings and to uncover the underlying biological and behavioral mechanisms involved in this bidirectional relationship.

## Data Availability

Publicly available datasets were analyzed in this study. This data can be found here: https://charls.pku.edu.cn/en/ The China Health and Retirement Longitudinal Study (CHARLS).
