# Supplementary material for: The longitudinal bidirectional association between cardiovascular disease and depressive symptoms among middle-aged and elderly adults: evidence from a nationwide cohort study in China
Source: Front Psychiatry. 2025 Jul 2;16:1559092. doi: 10.3389/fpsyt.2025.1559092 (PMC12263628; doi:10.3389/fpsyt.2025.1559092)
Supplement: Supplementary file 1 [file DataSheet1.docx]

**Supplementary Materials**

**Table S1. Hazard ratios (HRs) and 95% confidence intervals (CIs) for new-onset CVD associated with baseline depressive symptoms, among the participants without missing covariate data.**

| **Outcome** | **No. of event** | **Unadjusted model** | |  | **Partially adjusted model ^a^** | |  | **Fully adjusted model ^b^** | | |
| --- | --- | --- | --- | --- | --- | --- | --- | --- | --- | --- |
|  |  | HR (95% CI) | *P* |  | HR (95% CI) | *P* |  | HR (95% CI) | *P* | |
| **CVD** | 1590 | 1.64 (1.48, 1.82) | < 0.001 |  | 1.60 (1.42, 1.80) | < 0.001 |  | 1.60 (1.42, 1.80) | < 0.001 | |
| **CHD** | 1175 | 1.58 (1.40, 1.79) | < 0.001 |  | 1.55 (1.35, 1.77) | < 0.001 |  | 1.54 (1.34, 1.77) | < 0.001 | |
| **Stroke** | 544 | 1.86 (1.55, 2.22) | < 0.001 |  | 1.85 (1.51, 2.28) | < 0.001 |  | 1.85 (1.50, 2.27) | < 0.001 | |
| Abbreviations: HR, hazard ratio; CI, confidence interval.  ^a^ Partially adjusted model was adjusted for age, sex, BMI, residence, education level, marital status, alcohol consumption, smoking status, engagement in social activities, household fuel use, and personal earnings after tax  ^e^ Fully adjusted model was adjusted as the partially adjusted model with further adjustments for hypertension, diabetes, and dyslipidemia. | | | | | | | | | |  |

**Table S2. Hazard ratios (HRs) and 95% confidence intervals (CIs) for new-onset depression associated with baseline CVD conditions, among the participants without missing covariate data.**

| **Baseline condition** | **No. of new-onset depression** | **Unadjusted model** | |  | **Partially adjusted model ^a^** | |  | **Fully adjusted model ^b^** | | |
| --- | --- | --- | --- | --- | --- | --- | --- | --- | --- | --- |
|  |  | HR (95% CI) | *P* |  | HR (95% CI) | *P* |  | HR (95% CI) | *P* | |
| **CVD** | 2698 | 1.64 (1.48, 1.82) | < 0.001 |  | 1.60 (1.42, 1.80) | < 0.001 |  | 1.60 (1.42, 1.80) | < 0.001 | |
| **CHD** | 2698 | 1.58 (1.40, 1.79) | < 0.001 |  | 1.55 (1.35, 1.77) | < 0.001 |  | 1.54 (1.34, 1.77) | < 0.001 | |
| **Stroke** | 2698 | 1.86 (1.55, 2.22) | < 0.001 |  | 1.85 (1.51, 2.28) | < 0.001 |  | 1.85 (1.50, 2.27) | < 0.001 | |
| Abbreviations: HR, hazard ratio; CI, confidence interval.  ^a^ Partially adjusted model was adjusted for age, sex, BMI, residence, education level, marital status, alcohol consumption, smoking status, engagement in social activities, household fuel use, and personal earnings after tax  ^e^ Fully adjusted model was adjusted as the partially adjusted model with further adjustments for hypertension, diabetes, and dyslipidemia. | | | | | | | | | |  |

**Table S3. Hazard ratios (HRs) and 95% confidence intervals (CIs) for new-onset CVD associated with baseline depressive symptoms, by additionally adjusting PM_2.5_ exposure in the main Cox proportional hazards model.**

| **Outcome** | **No. of event** | HR (95% CI) | *P* |
| --- | --- | --- | --- |
| **CVD** | 1908 | 1.57 (1.42, 1.74) | < 0.001 |
| **CHD** | 1389 | 1.53 (1.36, 1.73) | < 0.001 |
| **Stroke** | 677 | 1.72 (1.43, 2.04) | < 0.001 |
| Abbreviations: HR, hazard ratio; CI, confidence interval.  The model was adjusted for age, sex, BMI, residence, education level, marital status, alcohol consumption, smoking status, engagement in social activities, household fuel use, personal earnings after tax, hypertension, diabetes, dyslipidemia, and long-term PM_2.5_ exposure level. | | | |

**Table S4. Hazard ratios (HRs) and 95% confidence intervals (CIs) for new-onset depression associated with baseline CVD conditions, by additionally adjusting PM_2.5_ exposure in the main Cox proportional hazards model.**

| **Baseline condition** | **No. of new-onset depression** | HR (95% CI) | *P* |
| --- | --- | --- | --- |
| **CVD** | 3162 | 1.23 (1.11, 1.36) | < 0.001 |
| **CHD** | 3162 | 1.20 (1.08, 1.34) | < 0.001 |
| **Stroke** | 3162 | 1.43 (1.16, 1.78) | < 0.001 |
| Abbreviations: HR, hazard ratio; CI, confidence interval.  The model was adjusted for age, sex, BMI, residence, education level, marital status, alcohol consumption, smoking status, engagement in social activities, household fuel use, personal earnings after tax, hypertension, diabetes, dyslipidemia, and long-term PM_2.5_ exposure level. | | | |


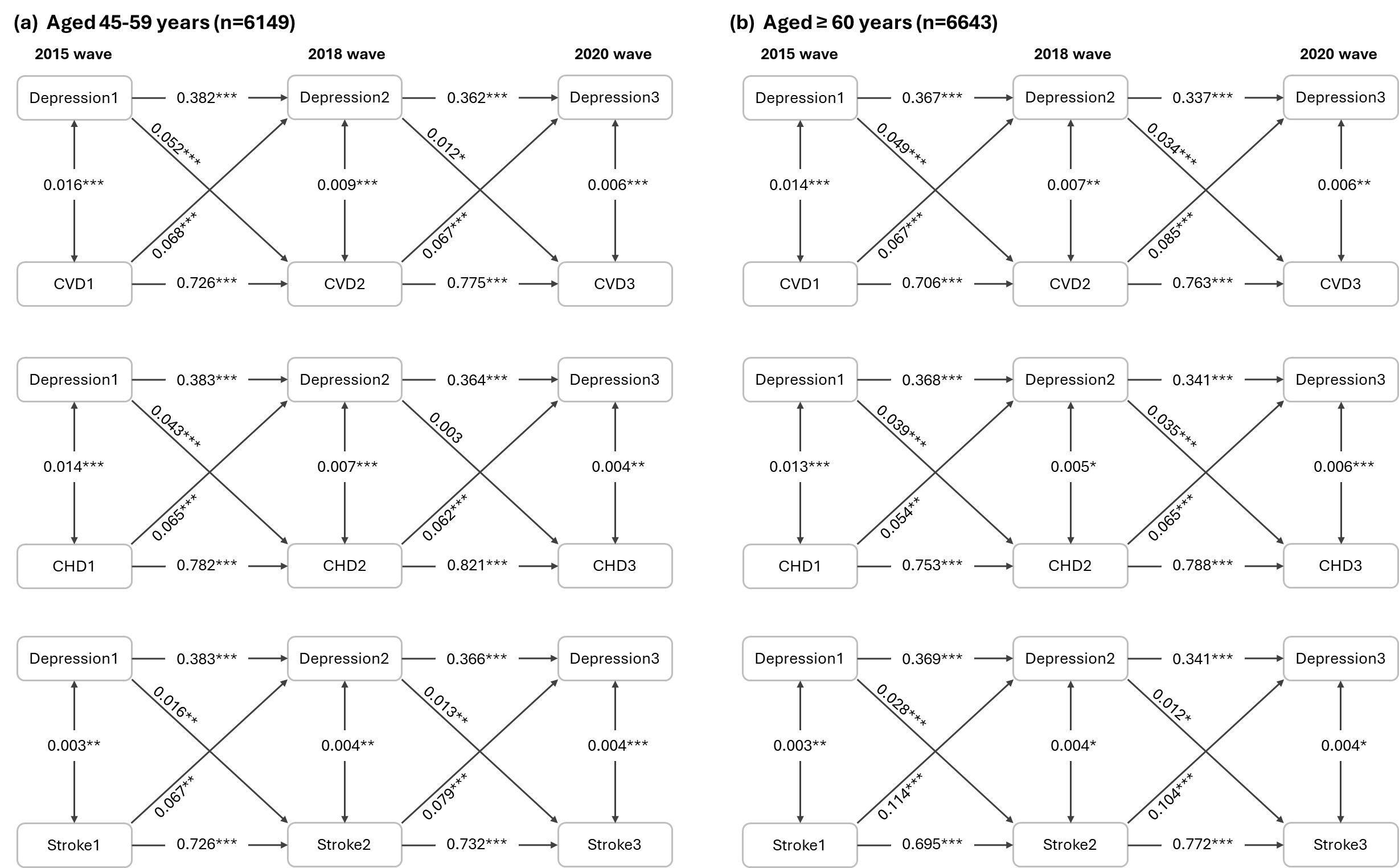


**Figure S1. Sensitivity analysis of CLPM among adults aged 45-59 years (a) and over 60 years (b).** All coefficients are standardized. Symbol * indicates 0.01 ≤ P < 0.05; Symbol ** indicates 0.001 ≤ P < 0.01; Symbol *** indicates P < 0.001. The model was adjusted for age, sex, BMI, residence, education level, marital status, alcohol consumption, smoking status, engagement in social activities, household fuel use, personal earnings after tax, hypertension, diabetes and dyslipidemia. Abbreviations: CHD, coronary heart disease; CVD, cardiovascular disease.


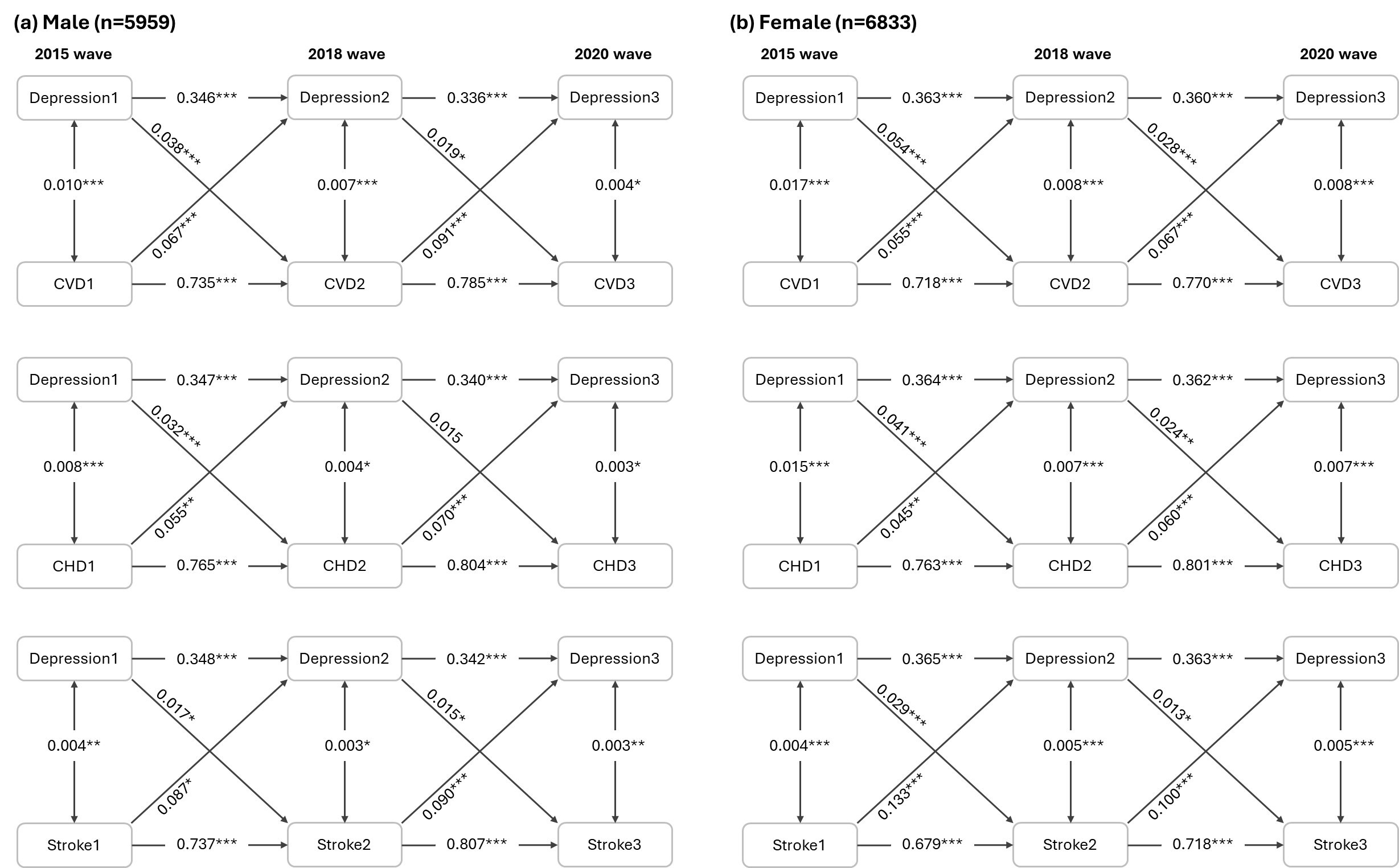


**Figure S2. Sensitivity analysis of CLPM among males (a) and females (b).** All coefficients are standardized. Symbol * indicates 0.01 ≤ P < 0.05; Symbol ** indicates 0.001 ≤ P < 0.01; Symbol *** indicates P < 0.001. The model was adjusted for age, sex, BMI, residence, education level, marital status, alcohol consumption, smoking status, engagement in social activities, household fuel use, personal earnings after tax, hypertension, diabetes and dyslipidemia. Abbreviations: CHD, coronary heart disease; CVD, cardiovascular disease.


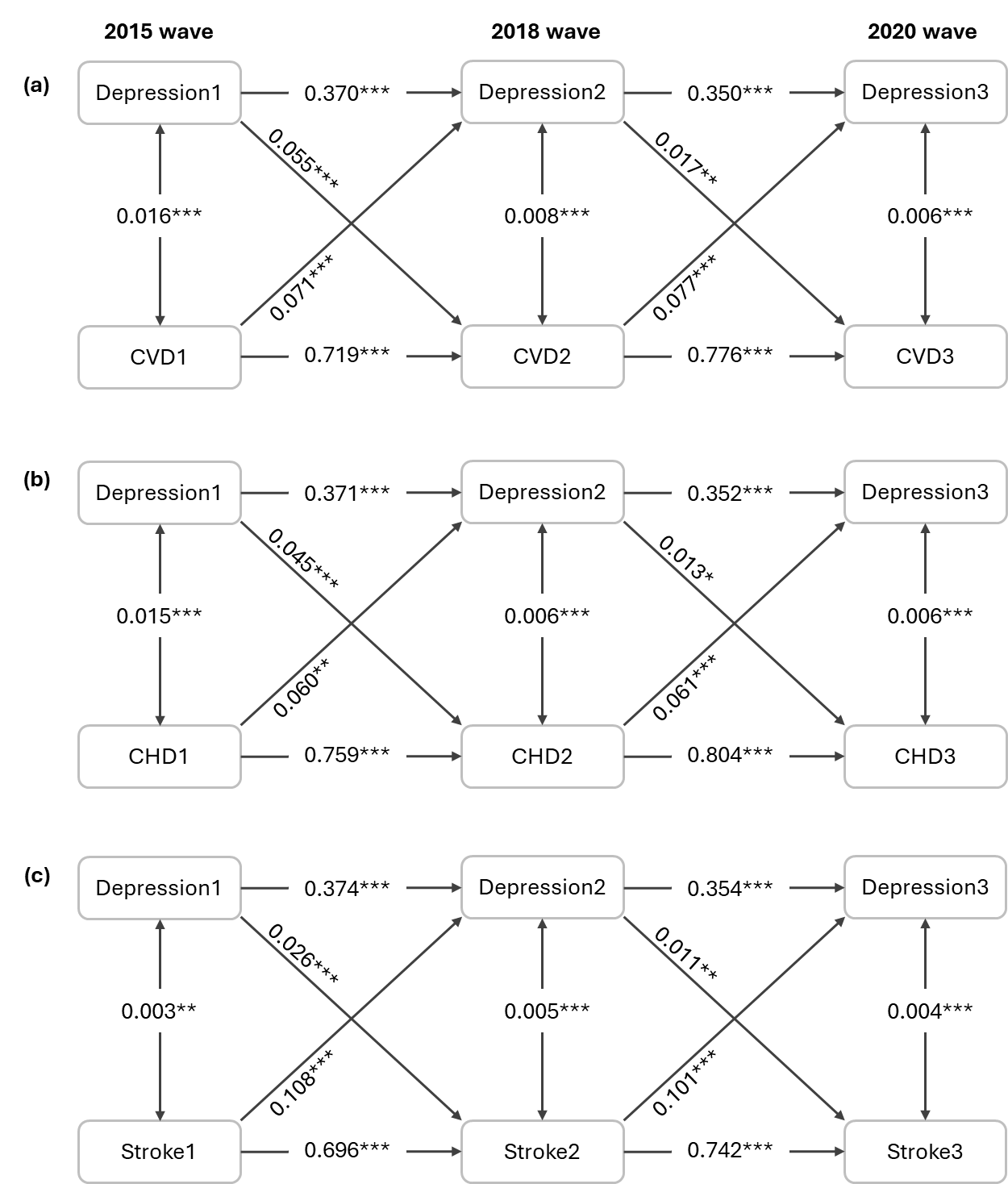


**Figure S3. Sensitivity analysis of CLPM by excluding participants with missing covariate data.** All coefficients are standardized. Symbol * indicates 0.01 ≤ P < 0.05; Symbol ** indicates 0.001 ≤ P < 0.01; Symbol *** indicates P < 0.001. The model was adjusted for age, sex, BMI, residence, education level, marital status, alcohol consumption, smoking status, engagement in social activities, household fuel use, personal earnings after tax, hypertension, diabetes and dyslipidemia. Abbreviations: CHD, coronary heart disease; CVD, cardiovascular disease.

**
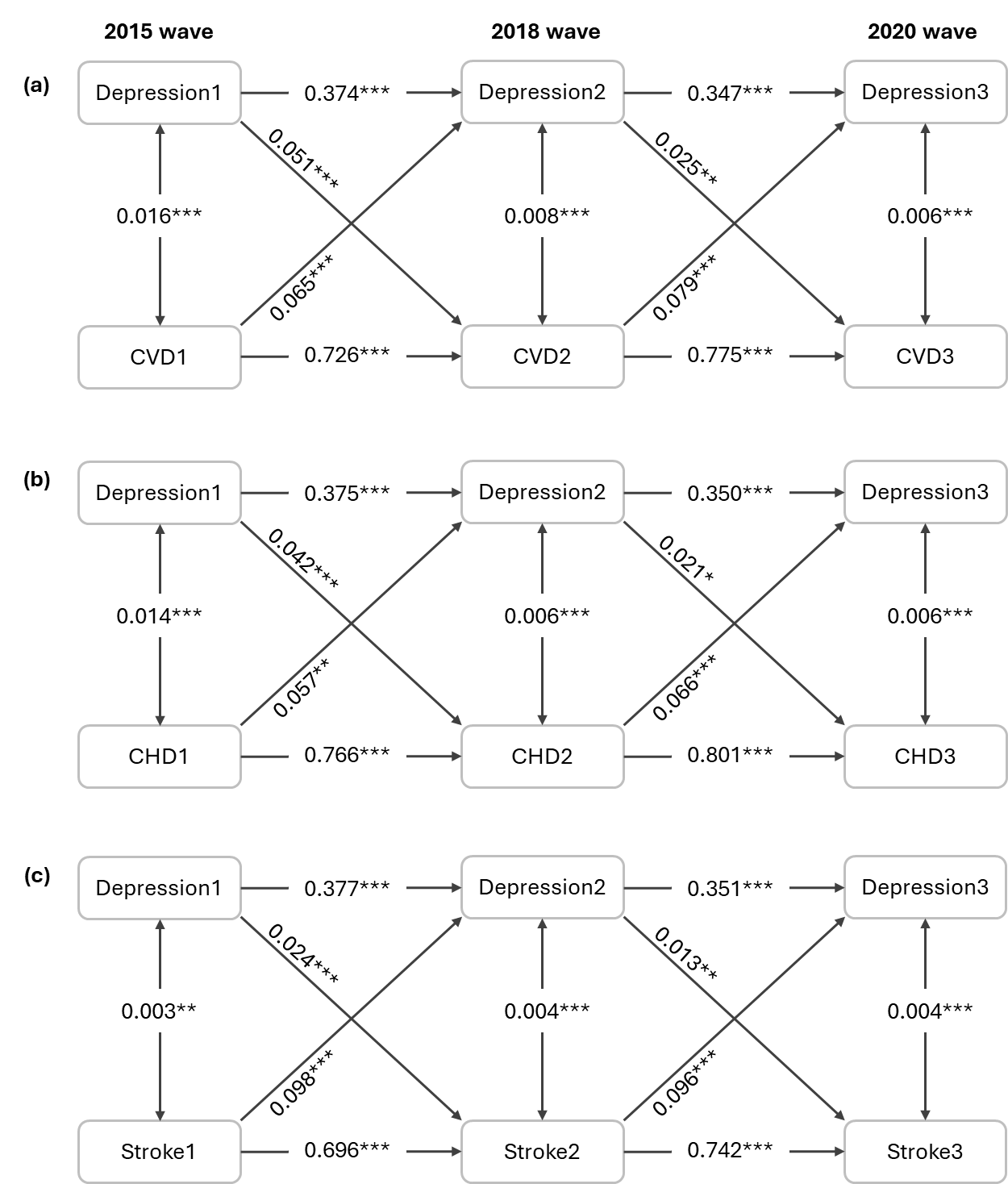
**

**Figure S4. Sensitivity analysis of CLPM by additionally adjusting for ambient PM_2.5_ exposure.** All coefficients are standardized. Symbol * indicates 0.01 ≤ P < 0.05; Symbol ** indicates 0.001 ≤ P < 0.01; Symbol *** indicates P < 0.001. The model was adjusted for age, sex, BMI, residence, education level, marital status, alcohol consumption, smoking status, engagement in social activities, household fuel use, personal earnings after tax, hypertension, diabetes and dyslipidemia. Abbreviations: CHD, coronary heart disease; CVD, cardiovascular disease.
